# Diagnostic and Prognostic Potentials of Long Noncoding RNA ELF3-AS1 in Glioma Patients

**DOI:** 10.1155/2020/8871746

**Published:** 2020-09-18

**Authors:** Jun-chi Mei, Ge Yan, Si-qing Mei

**Affiliations:** ^1^Department of Laboratory Medicine, The Renmin Hospital of Wuhan University, Wuhan, 410060 Hubei Province, China; ^2^Department of Laboratory Medicine, The Maternal and Child Hospital of Hubei Province, Tongji Medical College, The Huazhong University of Science and Technology, Wuhan, 410060 Hubei Province, China

## Abstract

**Objective:**

Accumulating evidence implies that long noncoding RNAs (lncRNAs) play a crucial role in predicting survival for glioma patients. However, the potential function of lncRNA ELF3-antisense RNA 1 (ELF3-AS1) in tumors remained largely unclear. The aim of this study was to explore the expression of lncRNA ELF3-antisense RNA 1 (ELF3-AS1) and evaluate its functions in glioma patients. *Patients and Methods*. ELF3-AS1 expressions were examined by RT-PCR in 182 pairs of glioma specimens and adjacent normal tissues. The receiver operating characteristic (ROC) curve was performed to estimate the diagnostic value of ELF3-AS1. The chi-square tests were used to examine the associations between ELF3-AS1 expression and the clinicopathological characters. The overall survival (OS) and disease-free survival (DFS) were analyzed by log-rank test, and survival curves were plotted according to Kaplan-Meier. The prognostic value of the ELF3-AS1 expression in glioma patients was further analyzed using univariate and multivariate Cox regression analyses. Loss-of-function assays were performed to determine the potential function of ELF3-AS1 on the proliferation and invasion of glioma cells.

**Results:**

The ELF3-AS1 expression level was significantly higher in glioma specimens compared with adjacent nontumor specimens (*p* < 0.01). A high expression of ELF3-AS1 was shown to be associated with the WHO grade (*p* = 0.023) and KPS score (*p* = 0.012). ROC assays revealed that high ELF3-AS1 expression had an AUC value of 0.8073 (95% CI: 0.7610 to 0.8535) for glioma. Using the Kaplan-Meier analysis, we found that patients with a high ELF3-AS1 expression had significantly poor OS (*p* = 0.006) and DFS (*p* = 0.0002). In a multivariate Cox model, we confirmed that ELF3-AS1 expression was an independent poor prognostic factor for glioma patients. The functional assay revealed that knockdown of ELF3-AS1 suppressed the proliferation and invasion of glioma cells.

**Conclusions:**

Our findings confirmed that ELF3-AS1 functions as an oncogene in glioma and indicated that ELF3-AS1 is not only an important prognostic marker but also a potential therapy target for glioma.

## 1. Introduction

Glioma is a common malignant primary brain tumor in adults and accounts for 35% of all central nervous system-related tumors as well as 85% of all primary malignant brain tumors^1^, ^2^. The existing WHO classification divides glioma into grades I, II, III, and IV from a histological perspective^3^. An upward trend in morbidity and mortality for glioma was observed from 2005 to 2018 in China^4^. Although new biological therapies have made progression with regard to the therapeutic intervention, including chemotherapy, radiotherapy, glioma surgery, gene therapy, and immunotherapy, glioma patients' overall survival (OS) is only 10-15 months after diagnosis^5^–^7^. On that account, it is necessary to develop new strategies for diagnosing and treating glioma, thereby reducing the recurrence as well as improving the OS.

Long noncoding RNAs (lncRNAs) are long RNA transcripts (>200 nucleotides) that do not possess protein-coding capabilities^8^. Different from short noncoding RNAs, lncRNAs exhibited an underestimated function role because of initially being identified as the transcriptional noise in the genome^9^, ^10^. Growing studies have indicated that lncRNAs participate in various biological activities, like cell differentiation, apoptosis, and gene expression epigenetic regulation, as well as RNA decay^11^, ^12^. Recently, aberrant lncRNA expressions are frequently found in most human cancers and affect each of the six hallmarks of cancer, and an increasing number of studies focus on revealing the molecular mechanisms exhibited by lncRNAs during the above-mentioned pathological processes^13^, ^14^. In addition, the development of high throughput sequencing promoted the detection technology of lncRNA, and according to the potential effects of lncRNAs acting as tumor promotors or suppressors, lncRNAs may serve as new diagnostic and prognostic biomarkers for various tumor patients, including glioma^10^, ^15^–^18^.

lncRNA ELF3-antisense RNA 1 (ELF3-AS1) was a recently identified lncRNA whose effects have been reported in various tumors, like lung cancer, osteosarcoma, and bladder cancer^19^–^21^. Interestingly, all previous studies have shown that the ELF3-AS1 expression was distinctly increased in various types of tumors. However, whether ELF3-AS1 also displayed a dysregulated expression in glioma and its biological significance have not been investigated.

## 2. Patients and Methods

### 2.1. Clinical Specimens

Between July 2012 and April 2015, 182 glioma samples together with paired adjacent noncancerous tissues were collected from glioma patients receiving surgical resection at our hospital. The five-year follow-up was completed specifically to all these patients, and their written informed consents were obtained. No patients had undergone previous treatments including radiation or chemotherapy. The inclusion criteria for the patient cohort included (i) having a distinctive pathological diagnosis of glioma; (ii) surgical resection, defined as the complete resection of all tumor nodules with the cut margin being free of tumors by histological examination; and (iii) having complete clinicopathological data. We obtained these samples during surgery and immediately froze them in liquid prior to use. Five-year follow-up was done through hospital medical records and telephone interviews. 172 glioma patients completed follow-up, and the rate of loss to follow-up was 5.4%.  Table 1 lists the clinical features exhibited by all these patients. This study has obtained the approval of the Research Ethics Committee of the Renmin Hospital of Wuhan University (No. WN33171). Written informed consent was obtained from all the patients.

### 2.2. Cell Culture and Cell Transfection

The human glioma cell lines (U251, H4, SW1783, and LN229) and NHA cells were obtained from a cell bank at the Chinese Academy of Sciences (Shanghai, China) and grown in Dulbecco's modified Eagle's medium (DMEM; Hyclone, Haidian, Beijing, China) containing 10% fetal bovine serum (FBS; Gibco, Hangzhou, Zhejiang, China) and 100 *μ*g/ml streptomycin and penicillin. All cell lines were maintained at 37°C in a humidified atmosphere containing 5% CO_2_.

### 2.3. RNA Extraction and Quantitative Real-Time PCR (qRT-PCR)

The TRIzol reagent (Invitrogen, Suzhou, Jiangsu, China) was employed to extract the total RNA. A PrimeScript 1st strand cDNA Synthesis Kit (TaKaRa Bio, Shenzhen, Guangdong, China) was applied to the synthesis of cDNA from total RNA relying on reverse transcription. The miScript SYBR Green PCR Kit (Qiagen, Haidian, Beijing, China) was applied to the ABI 7500 cycler (Applied Biosystems) for conducting the real-time PCR, based on the instruction of the manufacturer. The real-time PCR was conducted under the condition of 10 s at 94°C, 5 s at 94°C, 30 s of annealing at 52°C, and 15 s at 72°C followed by 40 cycles. The GenePharma company (Kunshan, Jiangsu, China) took charge of the design and synthesis of all these primers, as follows: ELF3-AS1, forward, 5′-GCAACGGCGTCTACCAC-3′; reverse, 5′-TAGCCCACGTCGTCTCACTATC-3′. GAPDH, forward, 5′-CGACTTATACATGGCCAAC-3′; reverse, 5′-TTCCGATCACTGGAATCAC-3′. GAPDH was used as an internal control. The relative level exhibited by gene expression was expressed relative to the GAPDH, and the 2^-*ΔΔ*Ct^ methods were adopted to calculate the relative level.

### 2.4. CCK-8 Assays

Cell proliferation was determined using CCK-8 (Haidian, Beijing, China). Cells were seeded in 96-well plates at a density of 2 × 10^3^ cells. After culture for 0, 24, 48, and 72 h, 10 *μ*l CCK-8 reagent was added to each well and incubated at 37°C for another 1 h. Optical density (OD) values were measured at 450 nm.

### 2.5. Colony Formation Assays

The capability of anchorage-independent growth of H4 and LN229 cells was measured by the colony formation assays. 1 × 10^3^ cells were plated in 10 cm dish and incubated in a humidified atmosphere of 5% CO_2_ incubator at 37°C for 10 days. For visualization, colonies were stained with 0.5% Crystal Violet (Sigma, Haidian, Beijing, China) in 50% methanol and 10% glacial acetic acid. The colonies with the diameters of >1 mm were counted.

### 2.6. Transwell Assays

Cells were transfected with 50 nM si-ELF3-AS1 or si-NC. Twenty-four hours postinfection, the infected cells were harvested and plated (1 × 10^5^) in the top chamber of transwell assay inserts (Millipore, Pudong, Shanghai, China) with a Matrigel-coated membrane containing 8 *μ*m pores. Inserts were then placed into the bottom chamber wells of a 24-well plate containing RPMI-1640 with 10% FBS as a chemoattractant. After 48 h of incubation, the remaining cells were removed from the top layer of the insert by scrubbing with a sterile cotton swab. Cells which passed through the filter were fixed and stained using 0.1% Crystal Violet. Numbers of invaded cells were counted in five randomly selected fields under a microscope.

### 2.7. Statistical Analysis

All data are represented as means ± SD. SPSS 19.0 software (SPSS Inc., Chicago, IL, USA) was applied to the analysis. Student's *t*-test together with the chi-square test assisted in estimating the difference significance between groups. The receiver operating characteristic (ROC) curves were aimed at discriminating glioma specimens from normal nontumor tissues. Kaplan-Meier methods together with a log-rank test assisted in determining the difference between patients in terms of the OS and DFS. Univariate and multivariate survival analyses adopted the Cox proportional hazard model. The values were considered to be statistically significant at *p* < 0.05.

## 3. Results

### 3.1. Expression Levels of ELF3-AS1 in Glioma

**Figure 1:**
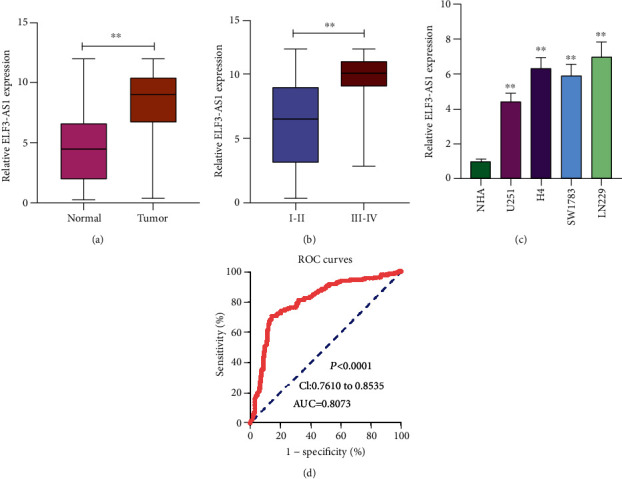
The expression of ELF3-AS1 and its diagnostic significance in glioma. (a) The expression levels of ELF3-AS1 in glioma tissues were significantly higher than those in corresponding noncancerous tissues. (b) RT-PCR for the determination of ELF3-AS1 expression in the glioma specimens with different stages. (c) qRT-PCR analysis of the expression of ELF3-AS1 in glioma cell lines and NHA cells. (d) ROC curve for diagnostic value of ELF3-AS1 in glioma. ^∗∗^*p* < 0.01, ^∗^*p* < 0.05.

To determine whether ELF3-AS1 was abnormally expressed in glioma, qRT-PCR assisted in examining the ELF3-AS1 expression exhibited by 182 pairs of glioma tissues as well as the noncancerous tissues.  Figure 1(a) demonstrates the obviously lower ELF3-AS1 expression in tumor tissues relative to noncancerous tissues (*p* < 0.01). In addition, we observed that the glioma specimens with advanced stages exhibited a higher level of ELF3-AS1 than those with early stages (Figure 1(b)). Moreover, we examined the levels of ELF3-AS1 in four glioma cell lines, finding that ELF3-AS1 expression levels were increased in four glioma cell lines compared with NHA, particularly in H4 and LN229 cells (Figure 1(c), *p* < 0.05). Accordingly, H4 and LN229 cells were used in the subsequent experiments. Thus, our findings revealed ELF3-AS1 as a regulator in the progression of glioma.

### 3.2. The Diagnostic Significance of Overexpression of ELF3-AS1 in Glioma

Previous studies have revealed that several functional lncRNAs displayed a diagnostic value in glioma patients. Then, we performed ROC assays which showed that high ELF3-AS1 expression had an AUC value of 0.8073 (95% CI: 0.7610 to 0.8535) for glioma (Figure 1(d)). The sensitivity and specificity of ELF3-AS1 expressions for distinguishing glioma samples from normal samples were 67.23%/85.22%, indicating ELF3-AS1 as an early-diagnosis indicator for glioma patients.

### 3.3. Association between ELF3-AS1 Expression and the Clinicopathological Features of Glioma

For a better understanding of the clinical relevance of the ELF3-AS1 expression in glioma, we divided the 182 glioma patients into a group with a high expression (*n* = 90) and a group with a low expression (*n* = 92), taking into account the median ELF3-AS1 expression level (5.232) in all glioma samples. Then, we performed a chi-square test and found that the high ELF3-AS1 expression affected the WHO grade (*p* = 0.023) and the KPS score (*p* = 0.012) (Table 1). However, there were no significant correlations of ELF3-AS1 expression with other clinical features.

### 3.4. Prognostic Values of ELF3-AS1 Expression as a Novel Biomarker in Glioma

**Figure 2:**
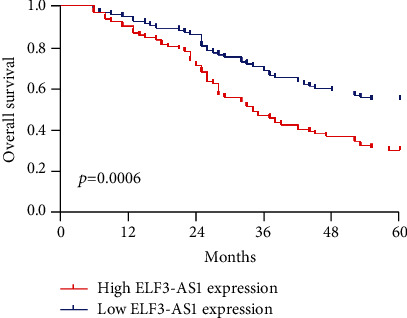
Kaplan-Meier curves estimating the 5-year overall survival rates according to the expression of ELF3-AS1 in patients with glioma.

**Figure 3:**
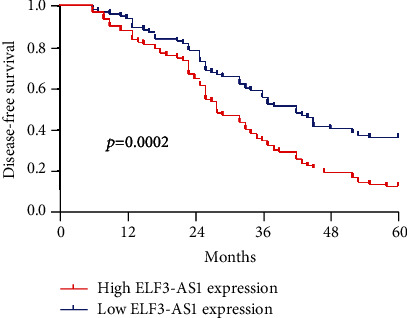
Kaplan-Meier curves estimating the 5-year disease-free survival rates according to the expression of ELF3-AS1 in patients with glioma.

Kaplan-Meier survival analysis assisted in confirming the association between the expression of ELF3-AS1 and 182 glioma patients' outcomes. Interestingly, we observed that patients who possessed a high ELF3-AS1 expression exhibited a weaker OS (*p* = 0.0006, Figure 2) and DFS (*p* = 0.0002, Figure 3) compared with those in the low ELF3-AS1 group. Moreover, univariate and multivariate assays were carried out to determine whether ELF3-AS1 was an independent factor for prognostic prediction in glioma patients. Importantly, our findings suggested that increased expression of ELF3-AS1 can be applied to independently predict the prognosis of patients regarding OS (HR = 2.673, 95% CI: 1.138-4.462, *p* = 0.015, Table 2) and DFS (HR = 2.762, 95% CI: 1.238-4.562, *p* = 0.006, Table 3).

### 3.5. The Effects of ELF3-AS1 Knockdown on the Proliferation and Invasion of Glioma Cells

**Figure 4:**
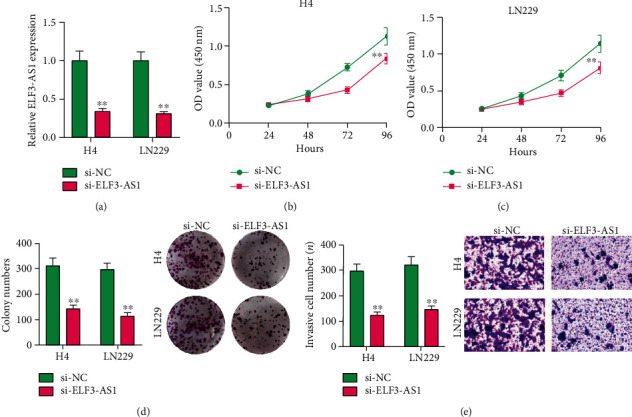
Effect of ELF3-AS1 on proliferation rate, colony formation ability, and invasion ability. (a) RT-PCR analysis of ELF3-AS1 knockdown efficiency in H4 and LN229 cells. (b, c) Cell proliferation was evaluated by CCK-8 assay in H4 and LN229 cells. (d) Colony-forming assays were performed to determine the growth of glioma cells. (e) Invasion ability was tested in Matrigel-coated transwell invasion chambers. ^∗∗^*p* < 0.01, ^∗^*p* < 0.05.

To determine the function of ELF3-AS1 in the progression of glioma, the expression of ELF3-AS1 was suppressed using si-ELF3-AS1 in H4 and LN229 cells (Figure 4(a)). CCK-8 assays showed that ELF3-AS1 silencing significantly inhibited the proliferation ability of glioma cells compared with that of negative control transfection (Figures 4(b) and 4(c)). The colony formation assay showed that H4 and LN229 cells transfected with si-ELF3-AS1 formed significantly less colonies than those transfected with si-NC (Figure 4(d)). Moreover, we explored the possible influence of ELF3-AS1 on the metastasis ability of glioma cells using transwell assays, finding that the cell invasion was markedly suppressed in glioma cells transfected with si-ELF3-AS1 as compared with cells transfected with si-NC (Figure 4(e)). Overall, our findings suggested ELF3-AS1 as a tumor promotor in glioma cells.

## 4. Discussion

Glioma acts as a common malignant primary brain tumor, and the significant progress of related treatment in the past ten years fails to improve its weak prognosis^22^, ^23^. The early screening and the prediction of clinical outcomes before various treatments may contribute to the optimization of therapeutic schedules for doctors, which then promoted the long-term survivals of glioma patients^24^, ^25^. Up to date, in clinical practice, the sensitive biomarkers were limited due to the unclear molecular mechanisms involved in the progression of glioma. In recent years, more and more studies have highlighted the potential of lncRNAs used as novel biomarkers for tumor patients due to their frequent dysregulation in both specimens and blood in patients and important effects in tumor progression^26^–^29^. In this study, we identified a novel glioma-related lncRNA, ELF3-AS1.

In recent years, the dysregulation of the ELF3-AS1 expression and its biological function in several tumors have been reported. For instance, Zhang et al.^21^ found that high ELF3-AS1 expression in lung cancer specimens predicted a weak clinical prognosis of lung cancer patients. Functionally, ELF3-AS1 was shown to promote lung cancer cells with regard to migration and invasion via sponging miRNA-212. Yuan et al.^20^ reported that ELF3-AS1, an overexpressed lncRNA in osteosarcoma, promoted the proliferation of osteosarcoma cells via increasing KLF12 potentially through methylation of miR-205. In bladder cancer, ELF3-AS1 was overexpressed and correlated with poor clinical outcome^19^. Moreover, in vitro and in vivo assays revealed that ELF3-AS1 knockdown hindered the proliferation and metastasis via interaction with KLF8. As suggested by another study of Chu et al., ELF3-AS1 overexpression promoted the proliferation of oral squamous cell carcinoma cell^30^. All the above findings suggested ELF3-AS1 as a tumor promotor. However, the expression and function of ELF3-AS1 in glioma have not been investigated.

Our study firstly proved the distinctly increased ELF3-AS1 in 182 glioma specimens compared to matched nontumor tissues, and increased ELF3-AS1 expression was associated with WHO grade and KPS score. Then, we explored the diagnostic value of a high ELF3-AS1 expression for glioma specimens, finding that a high ELF3-AS1 expression in the tumor specimens enabled the discrimination of glioma patients from nontumor brain tissues with an AUC of 0.8073, indicating it as a possible diagnostic biomarker for glioma. Moreover, we performed Kaplan-Meier methods to explore the clinical significance of ELF3-AS1 expression in glioma patients, finding that patients with a high ELF3-AS1 expression have a shorter OS and DFS than those with a low ELF3-AS1 expression. More importantly, the results of univariate and multivariate analyses confirmed that increased ELF3-AS1 expression could serve as a significant and independent predictor of OS and DFS of glioma patients. To explore the potential function of ELF3-AS1 on the progress of glioma cells, we performed loss-of-function assays and confirmed that knockdown of ELF3-AS1 suppressed the proliferation and invasion of glioma cells. The findings of the effects of ELF3-AS1 acting as a tumor promotor in glioma were consistent with previous findings in other types of tumors.

## 5. Conclusions

Our present study identified a novel glioma-related lncRNA, ELF3-AS1, which was associated with the progression of glioma. The findings revealed that ELF3-AS1 could be a new biomarker as well as a therapeutic target for effectively treating glioma.

## Figures and Tables

**Table 1:** Association between ELF3-AS1 expression and different clinicopathological features of 182 human gliomas.

Parameter	No. of cases	ELF3-AS1 expression		*p* value
		High	Low	
Age				0.667
<50	102	49	53	
≥50	80	41	39	
Gender				0.356
Male	105	55	50	
Female	77	35	42	
WHO grade				0.023
I–II	116	50	66	
III–IV	66	40	26	
KPS score				0.012
<80	80	48	32	
≥80	102	42	60	
Extent of resection				0.380
<98%	105	49	56	
≥98%	77	41	36	
Tumor size				0.103
<5 cm	106	47	59	
≥5 cm	76	43	33	

**Table 2:** Univariate and multivariate Cox regression analyses of ELF3-AS1 for overall survival of glioma patients.

Variable	Univariate analysis			Multivariate analysis		
	RR	95% CI	*p* value	RR	95% CI	*p* value
Age	0.842	0.452-1.623	0.241	—	—	—
<50 vs. ≥50						
Gender	1.213	0.773-1.898	0.423	—	—	—
Male vs. female						
WHO grade	3.113	1.342-4.782	0.008	2.893	1.234-4.345	0.015
I–II vs. III–IV						
KPS score	3.225	1.423-5.113	0.005	3.014	1.223-4.783	0.009
<80 vs. ≥80						
Extent of resection	0.892	0.472-1.885	0.231	—	—	—
<98% vs. ≥98%						
Tumor size (cm)	1.448	0.783-2.218	0.149	—	—	—
<5 vs. ≥5						
ELF3-AS1 expression	2.952	1.342-4.832	0.009	2.673	1.138-4.462	0.015
High vs. low						

**Table 3:** Univariate and multivariate Cox regression analyses of ELF3-AS1 for disease-free survival of glioma patients.

Variable	Univariate analysis			Multivariate analysis		
	RR	95% CI	*p* value	RR	95% CI	*p* value
Age	1.332	0.672-1.989	0.223	—	—	—
<50 vs. ≥50						
Gender	1.556	0.732-2.114	0.145	—	—	—
Male vs. female						
WHO grade	3.183	1.327-4.672	0.014	2.985	1.182-4.328	0.019
I–II vs. III–IV						
KPS score	2.987	1.247-4.558	0.017	2.632	1.124-4.134	0.021
<80 vs. ≥80						
Extent of resection	1.561	0.767-2.137	0.137			
<98% vs. ≥98%						
Tumor size (cm)	1.428	0.873-2.018	0.233	—	—	—
<5 vs. ≥5						
ELF3-AS1 expression	3.213	1.327-5.133	0.002	2.762	1.238-4.562	0.006
High vs. low						

## Data Availability

The data used to support the findings of this study are available from the corresponding author upon request.
